# Report from the Scientific Poster Session at the 13th Annual Cardiometabolic Health Congress in Boston, USA, 24–27 October 2018

**DOI:** 10.3390/medsci6040111

**Published:** 2018-11-30

**Authors:** Organizing Committee CMHC 2018

**Affiliations:** Tarsus Cardio dba Cardiometabolic Health Congress (CMHC), Boca Raton, FL 33431, USA; info@cardiometabolichealth.org; Tel.: +1-888-997-0112

## 1. Preface

More than one third of the population has at least one cardiometabolic risk factor-dyslipidemia, cardiovascular disease (CVD), hypertension, diabetes, and/or obesity. In its 13th year, the Cardiometabolic Health Congress (CMHC) is the largest, US-based, multidisciplinary conference focused solely on the management of cardiometabolic risk and the prevention of cardiovascular and metabolic disease and is chaired by top experts: Christie M. Ballantyne, MD; Robert H. Eckel, MD; George L. Bakris, MD; and Jay S. Skyler, MD. The three and a half-day event was attended by over 1000 cardiologists, endocrinologists, lipidologists and allied healthcare professionals from across the world and offered a one-of-a-kind opportunity to learn real-world solutions to integrate immediately into clinical practice.

The 2018 Cardiometabolic Health Congress was successful in providing up-to-date and clinically relevant education to clinicians. Several innovative and informative sessions were offered during the congress, including the pre-conference “Women’s Health Summit: Cardiometabolic Health Across the Lifespan,” which overviewed the intersection between breast cancer and cardiovascular disease, as well as the unique challenges faced by women in cardiometabolic health, including polycystic ovarian syndrome (PCOS), postmenopausal symptoms and management, contraception and adverse pregnancy outcomes.

The congress kicked off with the widely-popular Food and Drug Administration (FDA) Updates and Late Breaking Clinical Trials session where the attendees learned the latest developments in key cardiometabolic topics. Featured sessions highlighted an array of topics including triglyceride and low-density lipoprotein cholesterol (LDL-C) management, the evolving landscape of type 2 diabetes management, obesity and lifestyle medicine and new insights in the management of hypertension, heart failure and kidney disease. World-renowned speakers presented throughout the meeting, including keynote C. Ronald Kahn, MD; Deepak Bhatt, MD; Keith Ferdinand, MD; Irl Hirsch, MD; Peter Libby, MD; Anne Peters, MD; Paul M. Ridker, MD; Marc S. Sabatine, MD.

In addition to offering cutting-edge and comprehensive education, the 2018 CMHC hosted its second annual Scientific Poster Session, where investigators from around the world brought the latest data from current research and clinical findings to share with attendees.

## 2. Keynote Poster Abstracts

### 2.1. Cardioprotective Effect of Liraglutide Is Amplified with Anti-Inflammatory and Decreased Brain Natriuretic Peptide Levels, in Addition to Glycemia and Body Weight Reduction

PiljacAnte^1^JazbecAnamarija^2^DuvnjakLea Smircic^1^LjubicSpomenka^1^1Merkur Clinical Hospital, Vuk Vrhovac University Clinic, Zagreb, Croatia2University of Zagreb, Zagreb, Croatia

**Purpose:** Besides an impact on glycemic control and body weight, incretins emerged as important factors in cardiovascular (CV) protection in diabetes. Dipeptidyl peptidase-4 (DPP-4) inhibitors cleave multiple peptides, which in turn have direct effect on the heart and blood vessels. This distinguishes them in action when compared to glucagon-like peptide-1 (GLP-1) agonists. The aim was to compare the impact of DPP-4 inhibitors GLP-1 agonist liraglutide on CV risk factors.

**Methods:** A total of 442 type 2 diabetics were studied during a 6-month period and assigned into three study groups treated with DPP-4 inhibitors: linagliptin (Group (Gr) 1; *n* = 158), vildagliptin (Gr 2; *n* = 150) and with GLP-1 agonist liraglutide (Gr 3; *n* = 134). Adiponectin (ApN), brain natriuretic peptide (BNP), high specific C-reactive protein (hsCRP), blood pressure (BP), glycated hemoglobin (HbA1c) and other cardiovascular risk (CVR) factors were determined at the beginning and at the end of the follow-up period. Differences for the analyzed variables between baseline values and values after 6 months were tested by t paired test.

**Results:** Hs-CRP mean values at the beginning of the study were 3.86 ± 3.64, 2.67 ± 2.52 and 5.31 ± 2.37 in the Gr 1, 2, 3 respectively and were significantly reduced by 0.63 (95% CI: 0.1–1.15; *p* = 0.018), 1.35 (95% CI: −0.26–2.97; *p* = 0.09) and 1.71 (95% CI: 0.57–2.84; *p* = 0.007) on average in all three groups, with greater reduction in Gr 3 in comparison with Gr 2. HbA1c mean values at the beginning of the study were 8.01 ± 0.79, 7.36 ± 0.87 and 8.01 ± 0.95 in the Gr 1, 2, 3 respectively and were significantly reduced by 0.94 (95% CI: 0.73–1.15; *p* < 0.01), 0.69 (95% CI: 0.05–1.32; *p* = 0.04) and 1.15 (95% CI: 0.35–1.95; *p* < 0.01) on average, with no difference in reduction between groups. BNP and body mass index (BMI) were significantly reduced from baseline (30.5 ± 14.6 and 39.3 ± 4.5) by 10.7 (95% CI: 4.73–16.61; *p* = 0.002) and 2.65 (95% CI: 1.35–3.94; *p* < 0.01) on average in Gr 3, whereas reduction in systolic blood pressure (SBP) was significant from baseline (137.5 ± 16.9) in Gr 2 by 9.0 (95% CI: −0.05–18.55). Postprandial C-peptide, gamma-glutamyl transpeptidase (GGT) and triglycerides were reduced in Gr 3 by −0.32 (95% CI: −0.65–0.01; *p* = 0.058), 8.42 (95% CI: −0.15–16.9; *p* = 0.053) and 0.67 (95% CI: −0.09–1.43; *p* = 0.079) on average but these reductions were not significant. Increase in amylase was not observed in studied groups.

**Conclusions:** Except HbA1c and body mass index (BMI) reduction liraglutide proved more efficient in hs-CRP and BNP reduction in comparison with DPP-4 inhibitors. Treatment with liraglutide may exert cardioprotective benefits not only due to its glycemic control and body weight reduction but also through its pleiotropic effect.

### 2.2. Type 2 Diabetic Patients and Patients on Basal Supported Oral Therapy Mainly Benefit from Introduction of Insulin Degludec

LjubicSpomenka^1^,^2^PiljacAnte^1^JazbecA^2^AntalIvana^2^DuvnjakLea^1^,^2^1Merkur Clinical Hospital, Vuk Vrhovac University Clinic, Zagreb, Croatia2University of Zagreb, Zagreb, Croatia

**Purpose:** Decreased glucovariability of new insulins is one of the main reasons for better glycemic regulation, reduced incidence of hypoglycemia and body weight stability—parameters important in cardiovascular risk (CVR) reduction. Though latent autoimmune diabetes of the adults (LADA) may appear with diabetic ketoacidosis, it may also present in a mild non-insulin-requiring form. The potential value of screening patients with type 2 diabetes (DM2) for diabetes-associated autoantibodies to identify those with LADA is emphasized by similar clinical features, possibly worse glucose control than in DM2 and needs for a dedicated therapeutic strategy. The aim was to compare the efficacy and safety of long-acting insulin Degludec (DEG) in patients with DM2 and LADA after different therapy regimens.

**Methods:** After insulin DEG was introduced, 117 diabetic patients treated in our outpatient department were assigned to study groups according to the type of diabetes (DM2 and LADA) and previous therapy regimens (premix insulins 2–3 times daily (Gr 1), basal bolus (BB) therapy (Gr 2) and oral hypoglycemic agents (OHAs) (Gr 3)) and studied during a 6-mo follow-up. After changing premix insulins to BB therapy, DEG was introduced as basal insulin (BI); in patients on BB therapy previous BI was changed to DEG, whereas in patients on OHAs, DEG was added as a basal support (BOT). DM types were distinguished by determining islet cell (ICA) and glutamic acid decarboxylase autoantibodies (GADA). Body mass index (BMI), glycated hemoglobin (HbA1c), estimated average glucose (eAG), fasting blood glucose (FBG), lipids, albumin/creatinine ratio (ACR), glomerular filtration rate (GFR) and BI and bolus doses were determined at the beginning and at the end of the follow-up period.

**Results:** At the beginning of the study significant differences were observed in BMI (*p* = 0.02), HbA1c (*p* < 0.001), FBG (*p* = 0.014), eAG (*p* < 0.001), triglycerides (TG) (*p* = 0.041) and ACR (*p* = 0.041) among patient groups according to previous therapy regimens (ANOVA), whereas groups according to DM type differed significantly in BMI (*p* = 0.024), TG (*p* < 0.01), high-density lipoprotein (*p* = 0.017), ACR (*p* = 0.005) and GFR (*p* < 0.001) (Mann Whitney test). Six months after introduction of DEG, a statistically significant reduction in HbA1c (7.76 ± 1.09 vs. 7.01 ± 0.83), FBG (10.14 ± 2.97 vs. 8.26 ± 2.5) and eAG (9.76 ± 1.73 vs. 8.61± 1.3) (all *p* < 0.001) was observed (T paired test). According to previous therapy, mean HbA1c values were significantly reduced in all three groups (*p* < 0.001), with greater reduction in Gr 3 (8.82 ± 0.96 vs. 7.16 ± 1.2) compared to Gr 1 (7.91 ± 0.98 vs. 6.91 ± 0.76) and Gr 2 (7.35 ± 0.95 vs. 7.00 ± 0.73). Mean FBG values were significantly reduced in Gr 1 (11.37 ± 2.6 vs. 8.3 ± 1.42) (*p* < 0.001) and Gr 3 (11.26 ± 3.92 vs. 8.25 ± 3.15) but not in Gr 2 (9.14 ± 2.47 vs. 8.24 ± 2.75). Mean eAG values were significantly reduced in all three groups (Gr 1: 9.88 ± 1.42 vs. 8.42 ± 1.2; Gr 2: 9.15 ± 1.58 vs. 8.55 ± 1.21; and Gr 3: 11.44 ± 1.54 vs. 9.04 ± 1.69) (all *p* < 0.001), with greatest reduction in Gr 3 (Repeated measure ANOVA). According to DM type, mean HbA1c and eAG values were significantly reduced in both groups, with greater reduction in DM2 (HbA1c: 7.91 ± 1.07 vs. 7.0 ± 0.8; *p* < 0.001) compared to LADA. (HbA1c: 7.51 ± 1.18 vs. 6.98 ± 0.85; *p* = 0.03 and eAG 9.35 ± 1.9 vs. 8.53 ± 1.36; *p* = 0.04). Mean values of FBG (10.47 ± 2.89 vs. 8.2 ± 2.07) (*p* < 0.001) and TG (2.1 ± 1.58 vs. 1.91 ± 1.38) (*p* = 0.03) were significantly reduced in DM2 but not in LADA. Less hypoglycemic events were observed in 41 patients, mostly those with DM2 (28/41) and on BB therapy (33/41) after DEG introduction, with no increase in hypoglycemia incidence in 76 of the 117 studied patients.

**Conclusions:** Reduction in HbA1c, FBG and eAG after DEG introduction, especially in patients on BOT, suggests the benefit of early insulinization for the prevention of late diabetic complications. DM2 patients attained better HbA1c and eAG reduction during follow-up as compared with LADA. Reduction in hypoglycemia incidence after insulin DEG introduction suggests its beneficial effect on CVR reduction.

### 2.3. Cardiovascular Risk Reduction Associated with Pharmacological Weight Loss Therapy: A Meta-Analysis

KaneJesse A.MunirIrsaMehmoodTalhaKamranHaroonYacoubMenaYoussefIriniGustafsonDeborah R.McFarlaneSamy I.Sunny Downstate Medical Center, Brooklyn, NY 11203, USA

**Purpose:** Obesity is a growing pandemic that is associated with multiple cardiovascular disease (CVD) risk factors such as hypertension, diabetes mellitus, dyslipidemia and obstructive sleep apnea. With the rise of the pandemic of obesity, nearly two thirds of Americans are either obese or overweight and there has been an increase in the use of pharmacological therapy for the disease. While these therapies for weight loss have shown benefit in weight reduction, the clinical impact these medical therapies have on overall CVD outcomes has yet to be determined. We aimed to assess the effect of pharmacological agents used for weight reduction on CVD risk and all-cause mortality.

**Methods:** We conducted a systematic meta-analysis of peer-reviewed literature that evaluated the impact of anti-obesity drugs on CVD outcomes. Key words used included: “orlistat,” “lorcaserin,” “phentermine/topiramate” or “naltrexone/bupropione” and “cardiovascular outcomes” among others. We reviewed 791 articles, only 47 studies were randomized controlled trials and only 7 studies fulfilled all the inclusion criteria including, quantitative data on CVD risk factors such as, hemoglobin A1C (HbA1c), changes in body mass index (BMI), blood pressure and CVD morbidity and mortality. Data was retrieved from these studies and evaluated with comprehensive meta-analysis software to assess pooled effects for medical management versus placebo.

**Results:** There were a total of 7 studies included in the final analysis, this included a total of 18,598 subjects, of which 8685 were in the intervention (INT) group and 9913 in the control (CTRL) group. For all-cause mortality, there were 45 events in the INT and 55 in the CTRL groups, suggesting no significant difference between the two groups (OR: 0.843, 95% CI: 0.571–1.244, Z: −0.860, P: 0.390). For CVD mortality, there were 17 events in the INT and 36 events in the CTRL groups suggesting a significant mortality benefit in the INT group (OR: 0.496, 95% CI: 0.282–0.873, Z: −2.433, P: 0.015). There was a significant absolute reduction in A1C in the INT group (Hg: −0.238, 95% CI: −0.291–−0.186, Z: −8.937, *p* < 0.001). The percentage weight reduction was significantly higher for the INT group compared to the CTRL group (Hg: −0.431, 95% CI: −0.477–−0.385, Z: −18.472, *p* < 0.001) and the blood pressure reduction was higher for the INT group compared to the CTRL group. (Hg: −0.052, 95% CI: −0.101–−0.003, Z: −2.086, P: 0.037). The heterogeneity observed for our meta-analysis is Q: 1.884, df: 6, P: 0.930.

**Conclusions:** Our study demonstrated the favorable and significant effect of pharmacological weight reduction strategies on weight loss, blood pressure reduction, A1C reduction and CVD mortality. Given the limited efficacy of the lifestyle modification on sustained weight loss and the surgical risk and limited availability of bariatric surgical options, our data suggests pharmacological weight loss therapy may be a valuable treatment option to reduce CVD risk in obese patients. Further research should be performed to clarify the implications these therapies have on overall mortality and evaluate the mechanisms by which these medications reduce CVD risk factors and mortality.

### 2.4. The Effects of RYGB on Tissue Insulin Sensitivity, Beta Cell Function and Post-Meal Glucose Flux Are Maintained 7 Years after Surgery in Both Diabetic and Non Diabetic Patients

PalumboMaria^1^AstiarragaBrenno D.^1^BarbieriChiara^2^GagginiMelania^2^VecoliCecilia^3^ZampaVirna^4^AnselminoMarco^3^MariAndrea^2^GastaldelliAmalia^5^CamastraStefania^1^1Department of Clinical and Experimental Medicine, University of Pisa, Pisa, Italy2Institute of Clinical Physiology, CNR, Pisa, Italy3CNR Institute of Clinical Physiology, Pisa, Italy4Radiodiagnostic Unit, Santa Chiara Hospital, Pisa, Italy5Bariatric Surgery Unit, Santa Chiara Hospital, Pisa, Italy

**Purpose:** The improvement in Type 2 Diabetes (T2D) after Roux-en-Y gastric bypass (RYGB) is accompanied by change in insulin sensitivity (IS), β-cell function, post-meal glucose flux. Long-term studies indicate that the remission rate of T2D was higher at 2 that 10 years post-surgery. Most of the studies analyzed the effects of surgery on glucose metabolism in the early years after surgery Our aim was to determinate if the effects of surgery on tissue IS, post meal glucose flux and β-cell function (β-GS) are maintained long term after surgery.

**Methods:** We recalled 24 patients (14 T2D and 10 nondiabetic (ND)) that underwent RYGB 7 years earlier (7ys). In each patient we performed the same protocol that was performed before RYGB (B) and 1 year later (1y). The protocol consisted in a mixed meal test (MTT) and euglycemic-insulin-clamp combined with glucose and glycerol tracer to measure β-cell function (β-GS), glucose fluxes, adipose tissue insulin resistance (AT-IR), hepatic Insulin Resistance (H-IR), muscle IS (M/I).

**Results:** Both ND and T2D patients at 7ys regained 15 ± 6% of weight lost 1y after RYGB. T2D was resolved 1y post-surgery and this outcome was maintained at 7ys (HbA1c 56 ± 6 vs. 36 ± 1 vs. 41 ± 2 mmol/mol; B, 1y and 7ys). M/I improved at 1y (from 7.1 ± 1.5 to 13.5 ± 1.0 in ND and from 5.4 ± 0.8 to 13.8 ± 1.4 in T2D), *p* < 0.001 and maintained at 7ys in both ND and T2D (16.5 ± 2.5 in ND to 13.5 ± 1.5 nmol·min^−1^·kg_FFM_^−1^·pM^−1^ in T2D; *p* = ns vs. 1y). Hepatic-IR improved at 1y (from 0.9 ± 0.2 to 0.49 ± 0.08 in ND and 1.05 ± 0.2 to 0.72 ± 0.10 in T2D, *p* < 0.02) and maintained at 7ys (0.52 ± 0.10 in ND to 0.59 ± 0.12 nmol. kgffm-1. min-1. PM-1 in T2D; *p* = ns for both). The same results for AT-IR that improved at 1y (*p* = 0.03) and maintained at 7ys in both ND and T2D (*p* = ns). Plasma glucose profile and the dynamic of the oral glucose Ra were similar in ND and T2D at 1y and 7ys. Post meal suppression of endogenous glucose production during the first 90 min was improved in both groups at 7ys compared to 1y (AUC of EGP 0–90 *p* < 0.05, 1 yr vs. 7 yrs, for both group). In T2D, the improvement in β-GS seen at 1y (33 ± 5 to 64 ± 8 pmol·min^−1^·m^−2^·mM^−1^, *p* = 0.001) was maintained at 7ys (79 ± 15) at a similar level as in ND (136 ± 16 vs. 88 ± 8 vs. 83 ± 9, B, 1- and 7ys).

**Conclusions:** In both ND and T2D, RYGB induces marked improvements in glucose tolerance, insulin sensitivity (muscle, liver, adipose tissue) and β-cell function that are maintained 7 years after surgery.

### 2.5. Long-Term Effect of Patiromer for Hyperkalemia Treatment in Patients with HFmrEF and Diabetic Nephropathy on RAAS Inhibitors

PittBertram^1^MayoMartha R.^2^GarzaDahlia^2^ArthurSusan P.^2^LainscakMitja^3^1Department of Internal Medicine, University of Michigan School of Medicine, Ann Arbor, MI 48109, USA2Relypsa, Inc., a Vifor Pharma Group Company, Redwood City, CA 94063, USA3Division of Cardiology, General Hospital Murska Sobota, Murska Sobota, Slovenia

**Purpose:** Heart failure (HF) patients with mid-range ejection fraction (HFmrEF, 40–49%) are an important subgroup requiring further study. Renin-angiotensin-aldosterone system inhibitors (RAASi) have not been shown to reduce mortality in these patients but are often used to manage coexisting conditions, such as hypertension (HTN), diabetes mellitus (DM) and chronic kidney disease (CKD), or to provide symptom relief. Chronic hyperkalemia and CKD may complicate use of RAASi. Patiromer, a sodium-free non-absorbed potassium binder that uses calcium as the counter-exchange ion, is approved for the treatment of hyperkalemia, including in the US, the EU and Australia. The long-term effects of patiromer on serum potassium in HFmrEF patients on RAASi were examined in a post-hoc analysis of AMETHYST-DN.

**Methods:** Patients with CKD, type 2 DM and hyperkalemia (baseline potassium >5.0–<6.0 mEq/L) were randomized to patiromer starting doses 8.4–33.6 g/day, divided twice daily. Patients with HTN were required to have an average sitting systolic blood pressure (SBP): >130 to ≤180 mmHg; and diastolic blood pressure (DBP) >80 to ≤110 mmHg at screening. Patients remained on RAASi during study treatment. Changes in mean serum potassium (central lab) from baseline through 52 weeks were evaluated in the HFmrEF subgroup.

**Results:** 46/304 patients who were randomized and received at least 1 patiromer dose had HFmrEF (100% Caucasian, 74% male, 72% ≥65 years; mean [SD] EF = 44 [3] % and estimated glomerular filtration rate [eGFR] = 42 [14] mL/min/1.73 m^2^). All had HTN (baseline mean BP 154/84 mmHg). Mean serum potassium was reduced to <5.0 mEq/L at the rst post-baseline visit (day 3; 48 h after starting patiromer); from a mean (SE) value at baseline of 5.21 (0.06) mEq/L, the mean (SE) change from baseline on day 3 was −0.32 (0.06) mEq/L. Mean serum potassium was then maintained <5.0 mEq/L through week 52; the mean (SE) change from baseline in serum potassium at week 52 was –0.58 (0.1) mEq/L. From weeks 12 to 52, ≥85% of patients had serum potassium in the target range of 3.8 to 5.0 mEq/L at monthly visits. Thirty-three (72%) patients reported ≥1 adverse event (AE); influenza and worsening of CKD were the 2 most common AEs (5 patients each; none severe). Two patients had serum potassium <3.5 mEq/L; 1 patient had serum magnesium <1.2 mg/dL (none <1.0 mg/dL). Mean (SD) change from baseline to 52 weeks was: eGFR, +5 (19.6) mL/min/1.73 m^2^; SBP/DBP, −21 (19.2)/−10 (11.7) mmHg.

**Conclusions:** These post-hoc results suggest that patiromer allows control of hyperkalemia in HFmrEF patients on RAASi and require prospective evaluation.

### 2.6. Effect of Patiromer on Serum Potassium in Hyperkalemic Patients with and without Obesity: Pooled Results from the AMETHYST-DN, OPAL-HK and TOURMALINE Trials

RossignolPatrick^1^GrossColeman^2^MayoMartha R.^2^WarrenSuzette^2^YuanJinwei^2^BuddenJeffrey^2^MoralesEnrique^3^1Centre d’Investigations Cliniques-Plurithématique, INSERM, Université de Lorraine, Nancy, France2Relypsa, Inc., a Vifor Pharma Group Company, Redwood City, CA 94063, USA3Servicio de Nefrología, Hospital Universitario 12 de Octubre, Madrid, Spain

**Purpose:** Obesity (defined as body mass index (BMI) ≥ 30 kg/m^2^) is common in patients with chronic kidney disease (CKD). Activation of the renin-angiotensin-aldosterone system may play a causative role in in the development of CKD in obesity along with other factors such as proteinuria and hypertension. Renin-angiotensin-aldosterone system inhibitors (RAASi) are effective in CKD or heart failure patients, with or without obesity. The risk of hyperkalemia in CKD or heart failure treated with RAASi, including aldosterone antagonists, is similar in obese and non-obese patients. Patiromer, a non-absorbed, sodium-free, potassium binder, has been shown to effectively reduce serum potassium levels in hyperkalemic patients with CKD taking RAASi. The effect of patiromer in obese patients with hyperkalemia and CKD has not been previously reported.

**Methods:** We evaluated patiromer’s effect on serum potassium in hyperkalemic patients with and without obesity. The effect of four weeks of treatment with patiromer on serum potassium was investigated by combining data from three studies: AMETHYST-DN, OPALHK and TOURMALINE. Eligible patients had to have serum potassium (local lab) >5.0 mEq/L to receive patiromer. Starting doses of patiromer ranged from 8.4 to 33.6 g/d. Patients who took at least one dose of patiromer and had one post-baseline serum potassium measurement were included in this post-hoc analysis. Data were pooled and analyzed for change from baseline in serum potassium (primary endpoint in OPAL-HK and AMETHYST-DN; secondary endpoint in TOURMALINE) and the proportion of patients achieving target serum potassium (primary endpoint in TOURMALINE; secondary endpoints in OPAL-HK and AMETHYST-DN) according to BMI (≥30 kg/m^2^ obese and <30 kg/m^2^ non-obese) at baseline visit.

**Results:** Of 653 patients included in the analysis, 62% were men, the mean (SD) age was 66 ± 9.9 years and 40.9% were obese. Other than BMI, patient characteristics were similar among obese and non-obese patients including baseline potassium (mean [SD] 5.39 ± 0.38, 5.39 ± 0.44 mEq/L), diabetes mellitus (87%, 78%) and eGFR (mean [SD] 38.8 ± 19, 39.7 ± 19 mL/min/1.73 m^2^) and the presence of hypertension (99%, 97%). More than 90% of patients were on RAASi during the studies. More patients with obesity were receiving dual RAASi blockade (21.0% vs. 10.9%) and aldosterone antagonists (10.5% vs. 5.4%). At Week 4, least square mean (SE) serum change from baseline in potassium was −0.77 ± 0.03 mEq/L (*n* = 240) and −0.74 ± 0.03 in obese and non-obese patients, respectively. The proportion of patients who achieved a target range potassium (3.8–5.0 mEq/L) by Week 4 was 96% in obese patients and 97% in non-obese. In those with moderate hyperkalemia (potassium ≥5.5 mEq/L) at baseline, 95.4% of obese and 95.7% of non-obese patients achieved target serum potassium by Week 4. Adverse events (AEs) were reported in 101 (38%) obese patients and 108 (28%) non-obese patients; the three most common AEs in obese and non-obese patients were, respectively, constipation: 7.1% and 5.2%, diarrhea: 3.0 % and 2.8% and hypomagnesemia: 3.4% and 1.0%. Most AEs were mild to moderate in severity. AEs led to patiromer discontinuation in 4.1% of obese patients and 3.4% of non-obese patients.

**Conclusions:** Patiromer reduced serum potassium in hyperkalemic obese and non-obese patients in a manner consistent with overall results in the individual studies.

### 2.7. Association of Body Mass Index and Diastolic Function in Metabolically Healthy Obese with Preserved Ejection Fraction

RozenbaumZach^1^TopilskyYan^1^KhouryShafik^1^PeregDavid^2^Laufer-PerlMichal^1^1Department of Cardiology, Tel Aviv Medical Center, Tel Aviv, Israel2Department of Cardiology, Meir Medical Center, Kfar Saba, Israel*Dr. Pereg and Dr. Laufer-Perl equally contributed to the study.

**Purpose:** To characterize the relation between body mass index (BMI) and diastolic function in a relatively large cohort of metabolically healthy obese with preserved ejection fraction.

**Methods:** Echocardiograms of metabolically healthy patients between 2011–2016, who had no significant valvulopathies or atrial fibrillation and had preserved ejection fraction, were retrospectively identified and analyzed. Metabolically healthy was defined as lack of known diabetes mellitus, hypertension and hyperlipidemia. Patients were categorized into 4 groups according to BMI—normal BMI 18.5–25, overweight 25.01–30, obese 30.01–35, morbidly obese >35 kg/m^2^.

**Results:** The cohort consisted of 7057 individuals, 54.9% males, with a mean age of 54 years. Patients in higher BMI groups more commonly demonstrated abnormalities in most echocardiographic parameters associated with diastolic dysfunction, including left atrial volume index >34 mL/m^2^, E/e’ >14, e’ lateral <7 cm/s, tricuspid regurgitation velocity >2.8 m/s and systolic pulmonary artery pressure ≥36 mmHg (*p* < 0.01 for all comparisons). Morbidly obese carried the highest risk compared to those with normal BMI. There were no significant differences between the groups in rates of readmission due to heart failure.

**Conclusions:** High BMI is associated with increased risk of diastolic dysfunction even in metabolically healthy patients. Additional trials are needed in order to evaluate whether these echocardiographic findings translate into clinical implications.

### 2.8. Results of Intensive Weight Loss Program (IWLP) (Non-Surgical) in a Primary Care Office. Comparison of IWLP and CDC Diabetes Prevention Program (CDC DPP)

AhmedMahtab^1^RaderAllen^2^1Activate Healthcare—Cooper Farms, Van Wert, OH 45891, USA2Idaho Weight Loss, Boise, ID 83703, USA

**Purpose:** Patients and primary care physicians (PCPs) face difficulties in knowledge, motivation, expectation, appropriate setting and affordability of services for a user-friendly effective weight loss and maintenance program in a primary care office. The key concepts of weight management such as behavioral, nutritional, physical activity and pharmaceutical interventions requires resources which is challenging for the primary care office. It is well established that ten percent weight loss helps in reduction of cardiovascular (CVD) risk. Health insurance coverage for nonsurgical obesity care is inadequate nationwide. Individuals in the national Centers for Disease Control–Diabetes Prevention Program (CDC-DPP) were able to achieve only 5% weight loss in 35 percent of participants. Our poster provides data from an individualized Intensive Weight Loss Program (IWLP) to incorporate behavioral, nutritional, physical activity and pharmaceutical interventions to achieve weight loss in a user friendly, medically safe environment in an employer based primary care clinic with one provider and two support staffs in Ohio. Biometrics and labs values and CVD risk were calculated and shows improvement in all parameters with weight loss and maintenance.

**Methods:** Patients were selected who were willing to participate in weight loss program and have a body mass index (BMI) > 30 or BMI > 27 with comorbid conditions such as hypertension, diabetes, prediabetes, hyperlipidemia, sleep apnea, GERD and arthritis. Patients were not eligible to participate who had BMI < 25, was pregnant or actively trying to get pregnant and patients with active cardiac diseases, major surgeries, gout, or cancer, psychiatric issues not controlled by treatment, current use of illegal drugs and history of dependence on alcohol, narcotics and controlled substance. 

Behavioral components of IWLP include daily body weight check and changing eating habits to 3 meals a day with an adequate amount of water. Nutritional components include either a low-calorie diet (<1200 calorie/day) or reducing carbohydrates to 60 gram/day and adding an adequate amount of protein (30 gram three times/day). Physical activity goal was 150 min per week (30 min/day for 5 days) or doubling up the daily step count from the baseline. Pharmacological components include multivitamins, Vitamin D and Fish Oil. Metformin, Phentermine (3 months only in Ohio) and Topiramate were used in selected patients with insulin resistance, binge eating and eating disorder respectively. 

The initial goal of IWLP was a 5% to 10% body weight loss in 3 to 6 months and to maintain the lost weight. Participants were required to see the physician monthly, starting with an initial H & P and coaching. They were offered an optional weekly or biweekly visit with the nurse for weight and vitals check for 3 months After finishing the 12-week program, further goals and appointments were individualized according to patient’s need. Patients were advised to keep their daily weight, exercise and food intake logs. 

**Results:** Weight loss data from 78 adults (51 females, 27 males, 23 to 63 YO) in a 7 to 92-week period (Average participation 41 weeks) is presented. Body weights presented here are initial, lowest and final body weight with corresponding weeks in each individual participant. 

“High risk group patients” (Male#18 Female#38 Total 56) were classified by using following criteria: waist circumference, BMI, high-density lipoprotein (HDL), glycated hemoglobin (HbA1c) and triglyceride (TG) (at least 3 out of 5 criteria).

Maximum weight loss ranged from 7.2 to 130 pounds with average weight loss 30.3 pounds, corresponding to 4.6% to 35.7% weight loss with average 12.5% weight loss. Final weight loss ranged from 1.6 to 130.4 pounds with average weight loss 24 pounds, corresponding to 0.9% to 35.7% weight loss with average 9.8% weight loss. Our data shows that 43% of participants lost more than 9.9% of body weight and maintained. 83% of participants lost more than 4.9% of body weight and maintained. 17% of participants lost less than 4.9%. None of the patients regained all the lost weight. Average waist circumference, HbA1c, total cholesterol, TG, low-density lipoprotein (LDL) were reduced and HDL were elevated with weight loss. “High risk group” showed similar changes with weight loss. Average Framingham scores were reduced (7.7–4.8) = 38% and (1.8–1.5) = 17% in male and female group respectively. Average ACC-AHA scores were reduced (11.4–8.6) = 24.6% and (4.2–3.4) = 18% in male and female respectively. “High risk group” lost average 30.9 pounds (10.6%) in male and 32 pounds (13.5%) in female groups. Average Framingham scores were reduced (7.88–5.85) = 25.8% and (1.6–1.41) = 11.9% in male and female group respectively. Average ACC-AHA scores were reduced (11.72–11.37) = 2.9% and (4.27–3.58) = 16.1% in male and female respectively. 

IWLP were compared with CDC DPP as follows: Average weeks 41 vs. 208, number adults 78 vs. 14,474, # of visits 1 visit/30 days vs. 1 visit/12 days, # of participants achieved >4.9% weight loss 83% in IWLP vs. 35.5% in CDC DPP. Average weight loss was 9.8% in IWLP vs. 4.2% in CDC DPP.

**Conclusions:** This poster demonstrates that a primary care physician with current knowledge in obesity management using the Obesity Algorithm from the Obesity Medicine Association should be ready to offer individualized cost-effective program for weight management with minimal support staff and equipment. There was improvement in biometrics (Body weight, BMI, Waist circumference); labs values (HbA1c, Total cholesterol, Triglycerides, HDL and LDL), Framingham score and ACC-AHA risk scores, thus in overall health and wellness.

### 2.9. Lowered Glucose Levels and Exogenous Insulin Requirements in T2DM and T1DM Patients Treated with Oral Insulin (ORMD-0801): Phase 2, Randomized, Placebo-Controlled Evaluations

KidronMiriam^1^HomerKeneth^2^NeutelJoel^3^1Oramed Pharmaceuticals, Jerusalem, Israel2Integrium LLC, Cedar Knolls, NJ 07927, USA3St. Joseph Hospital, Tustin, CA 92780, USA

**Purpose:** Consensus regarding the convenience of oral insulin, alongside the proposed therapeutic advantages of this administration route over systemic exposure, have fueled numerous attempts at design of such a formulation. Portally infused insulin brings to more rapid and pronounced suppression of hepatic glucose production and to reduced circulating peripheral insulin levels as compared to systemically administered insulin. Oral insulin deposited directly into the portal vein is expected to have similar salient effects. Oramed Ltd. has developed an oral insulin formulation (ORMD-0801), which harnesses excipients to both hinder proteolysis in the small intestine and enhance translocation of insulin across the gut epithelial lining. Once transported across the gut wall, the insulin is ferried to the hepatic portal vein, mimicking the natural route of endogenous pancreatic insulin and then subjected to first-pass metabolism in the liver, before being delivered to peripheral sites of action. The pharmacokinetic/pharmacodynamic (PK/PD) profile of ORMD-0801 is well suited for the control of fasting blood glucose due to the delayed onset of action. Therefore, Oramed is pursuing the bed-time oral administration of ORMD-0801 for the treatment of elevated fasting blood glucose in adult patients with type 2 diabetes mellitus (T2DM). In parallel, the drug has been shown to minimize glucose instability when provided as an adjunct to subcutaneous insulin regimens in type 1 diabetes mellitus (T1DM) patients. These two prospective, randomized, placebo-controlled Phase II studies aimed to evaluate the impact of ORMD-0801 on blood glucose homeostasis in T2DM patients and to evaluate its impact on bolus insulin requirements in T1DM patients.

**Methods:** In a Phase 2a study, the effect of pre-prandial ORMD-0801 on exogenous insulin requirements was assessed in T1DM patients monitored with a continuous glucose monitor (CGM) over a 7-day double-blind treatment period. A single ORMD-0801 capsule (8 mg insulin) was administered three times daily, 45 min before meals to 15 patients, while 10 received placebo. Insulin requirements were documented and glucose levels were recorded with a blinded continuous glucose monitor. Similarly, in a Phase IIb randomized (1:1:1), double-blind, placebo-controlled, multicenter (*n* = 33) study, 192 adult patients with T2DM, participated in a 14-day placebo run-in period, followed by a 28-day treatment period with 16 mg ORMD-0801, 24 mg ORMD-0801 or placebo, self-administered at bedtime. Glucose levels were monitored, via a blinded CGM, during the last 7 days of the run-in and treatment periods.

**Results:** On all treatment days, ORMD-0801-treated T1DM patients showed consistently lower fasting plasma glucose (FPG) levels as compared to baseline, peaking at −60.2 mg/dL on day 7, versus a mere −10.2 mg/dL change measured for the placebo cohort at the same time point. Reduced FPG levels directly correlated with reduced rapid-acting insulin requirements, reaching a mean difference of −5.9 mIU/mL insulin intake between active versus placebo-treated patients on day 7. On day 7 of treatment, an equal number of hypoglycemic events (<60 mg/dL) requiring clinical intervention was reported for each cohort. In T2DM patients, the active treatment proved safe, well-tolerated and nonimmunogenic, with no serious adverse drug-related events reported. No significant difference in incidence and types of adverse events, including hyper/hypoglycemia, was noted between cohorts. CGM data indicated a significantly smaller change from baseline in nighttime glucose levels in the pooled ORMD-0801 (1.7 mg/dL) as compared to the placebo (13.7 mg/dL, *p* = 0.027) cohort. Mean 24-h glucose readings remained stable among ORMD-0801-treated patients (mean difference: −0.32 mg/dL), whereas patients receiving placebo demonstrated a mean 13.26 mg/dL change from baseline in these readings (*p* < 0.001). Similarly, mean change from baseline in fasting (5AM7AM) and daytime (6AM-10PM) CGM glucose were significantly smaller among ORMD-0801-treated patients as compared to those treated with placebo (−0.4 mg/dL vs. 16.0 mg/d (*p* < 0.001) and 0.9 mg/dL vs. 11.9 mg/dL (*p* < 0.001) respectively). In parallel, the mean change from baseline in HbA1c levels in the combined ORMD-0801 cohorts (−0.01%) was significantly smaller as compared to the placebo cohort (0.2%; *p* = 0.01) and was projected to show a 0.5% drop from baseline following 12 weeks of treatment.

**Conclusions:** Pre-prandial ORMD-0801 reduced the exogenous short-acting insulin demands required to maintain euglycemia in T1DM patients. In parallel, the active treatment led to a greater drop in FPG concentrations, when compared to placebo treatment, seemingly due to improved hepatic insulinization and subsequently normalized gluconeogenesis/glycogenolysis ratios. Similarly, ORMD-0801 treatment elicited a sustained and highly significant reduction in mean nighttime, fasting, daytime and 24-h glucose concentrations. In both patient populations, the treatment proved safe for use and well tolerated at the tested regimen.

### 2.10. Assessment of Cardiovascular Disease Risk and Therapeutic Patterns Among Urban Black Rheumatoid Arthritis Patients

McFarlaneIsabel M.BhamraManjeet S.TaklasinghNicholasKaplanIanDellingerElaineSmerlingJonathanPaltooKarenLenisDianaGondalIrfanTrevisonnoMichaelKollaSriSunny Downstate Medical Center, Brooklyn, NY 11203, USA and NYC Health + Hospitals Kings County, Brooklyn, NY 11203, USA

**Purpose:** Patients with rheumatoid arthritis (RA) have nearly twice the risk of cardiovascular disease (CVD) compared to the general population. Besides the traditional risk factors for CVD including obesity, diabetes, hypertension and dyslipidemia, patients with RA also have increased risk due to chronic inflammation and elevated cytokine levels.

Specialized CV risk models for RA that include disease activity measures, disability index, duration of disease, steroid use in addition to traditional risk factors have been proposed to accurately predict CVD in RA. While older therapeutic agents such as corticosteroids and nonsteroidal anti-inflammatory drugs (NSAIDs) increase CVD risk, modern therapy including disease-modifying antirheumatic drugs (DMARDs) and biologics have been shown to decrease CVD in RA populations.

We aim to assess the prevalence of CVD risk factors including traditional (obesity, hypertension, diabetes, dyslipidemia and smoking) as well as non-traditional ones (inflammatory markers, length of disease and disease severity among) in our RA predominantly Black population; the study will also examine the therapeutic patterns, compared to a predominately White population of the Consortium of Rheumatology Researchers of North America (CORRONA).

**Methods:** Retrospective study of patients ≥18 years old with a principal or secondary discharge diagnosis of RA identified by ICD-9 and ICD-10 codes. Records reviewed between 1/2010 to 5/2017 from two large NYC hospitals with predominantly Black population. Two independent investigators reviewed the cases identified by ICD-codes to confirm RA diagnosis by physician notes and the presence of DMARDs in the medication list or DMARD prescription. 

Cases were excluded for insufficient data for RA diagnosis with no current or past DMARD therapy and/or non-RA diagnosis of arthritis. Data abstraction was performed for the selected cases, utilizing the predesigned data collection sheet for the study. Data collection included demographics, smoking history, year of diagnosis, comorbidities including CVD, laboratory data, hand imaging and treatment regimens. Collected data was verified by a second investigator. Bilateral hand imaging findings are being reviewed utilizing the Simple Erosion Narrowing Score by musculoskeletal radiologist. 

Descriptive statistics was applied. We used measures of central tendencies and dispersion for continuous variables and frequency distribution for categorical variables. Data are presented as the mean ± standard deviation (±SD). We compared our predominantly Black RA population to previously published RA data with predominantly White cohorts; CORRONA to assess differences in CVD and CVD risk prole and features of RA disease severity as well as therapeutic patterns including the use of steroids, DMARDS and biologics.

**Results:** Of the 1142 RA patients identified by ICD codes, only 500 were confirmed as RA cases and included in this analysis. Mean age was 64.6 ± 14.8 (±SD), 87.8% were women, 83.4% were Black and 9.2% Hispanics. BMI was 28.8 ± 7.5 with 37% of the patients having BMI ≥ 30 (Kg/m^2^). 

Our predominantly Black (83.4%) cohort with RA duration of 13.1 ± 9.7 years was compared to predominantly White (89%) CORRONA cohort with RA duration of 10.1 ± 9.8 years. There were higher rates of CVD risk factors: hypertension (66.4% vs. 29%), dyslipidemia (41% vs. 25%), diabetes (28.0% vs. 8%) for our cohort compared to CORRONA respectively. Our cohort had lower rate of smoking (29.5% vs. 34%), compared to CORRONA cohort. Myocardial infarction or known coronary artery disease (19.4%) was similar to that reported in the CORRONA study. The rate of other CVD in our cohort that were not reported in the CORRONA study were: congestive heart failure (14.8%), stroke or transient ischemic attack (10.2%) and atrial fibrillation (8.4%).

In our study, erythrocyte sedimentation rate (ESR) was 62.4 ± 37.2 mm/hr. and 76.2% had C-reactive protein (CRP) >4 mg/L. Serological markers: Rheumatoid factor (RF)+ 75.5%, anti-citrullinated peptide antibodies (ACPA)+ 69.3%, RF+ or ACPA+ 85.8% (77% in the CORRONA cohort) and dual RF-ACPA+ in 54% of the patients. Utilizing SENS scoring (not reported in the CORRONA study); hand X-rays revealed: periarticular osteopenia, joint space narrowing and joint erosions in 96.6%, 72.2% and 67.8% respectively. Erosion score (maximum 32) was 12.6 ± 11.7 and joint space narrowing score (maximum 30) was 20.6 ± 11.9.

Prednisone was used in 56% (30% for the CORRONA population) with average dose 8.14 ± 17.5 mg/day, 22% (61% for the CORRONA study) on NSAIDs, 40% (84% for the CORRONA cohort) on methotrexate (average dose 6.6 ± 8.5 mg), 42.5% on other DMARDs and 16% (56% for the CORRONA patients) were on biologics. 

**Conclusions:** This is the first study of CVD in blacks with RA including assessment of disease severity and therapeutic patterns compared to Whites. We observed higher rates of CVD risk factors including obesity, diabetes, hypertension, dyslipidemia, compared to the White cohort of the CORONA study. Our population had aggressive disease with high rates of seropositivity, joint narrowing/erosions and elevated inflammatory markers. 

Our RA black cohort had nearly double the rate of steroid use (a risk factor for CVD) and less than one third utilization of biologics, (which lowers the risk of CVD risk), compared to Whites of the CORRONA study.

### 2.11. Differences in the Management of Acute Coronary Syndromes by Race and Proximity to Care in an Integrated Healthcare Delivery System in Northern Nevada 

RowanChristopher^1^MetcalfJim^1^GrzymskiJoseph^1^MuesKatherine E.^2^YedigarovaLarisa^2^WooClaudine^3^WilliamsKim A.^4^1Renown Heath, Reno, NV 89502, USA2Amgen Inc., Thousand Oaks, CA 91320, USA3UC Berkeley School of Public Health, Berkeley, CA 94720, USA4Rush University Medical Center, Chicago, IL 60612, USA

**Purpose:** Differences in cardiovascular treatment patterns among underserved minority populations have been observed, however, the patterns of care following an episode of acute coronary syndrome (ACS) based on race and an urban/rural residence have not been well described. Among a cohort of individuals with an episode of ACS who sought care within the Renown Health integrated healthcare delivery system in northern Nevada, we sought to describe patterns of follow-up care for Caucasians, non-Caucasians, urban patients who reside less than 100 miles from the primary regional medical center and rural patients who reside greater than 100 miles from the primary regional medical center.

**Methods:** Data from an 11-year Epic Electronic Medical Record (EMR) were utilized to identify 4,076 patients with an ACS event between 2007 and 2017 based on an inpatient International Classification of Diseases-Ninth Revision (ICD-9) or ICD-10 code. Patients were required to have at least one provider encounter in the year prior to and the year following the ACS index event. Baseline and follow up labs, medication prescriptions and clinic encounters were quantified.

**Results:** Among 3527 (86.5%) Caucasians and 549 (13.5%) non-Caucasians, the mean age was 69 and 64 years, respectively. Comparing Caucasians to non-Caucasians, diabetes (29% vs. 41%) and chronic kidney disease (CKD) (33% vs. 37%) were less frequent among Caucasian patients prior to the ACS index event. The mean baseline LDL-C was 85 mg/dL and 88 mg/dL among Caucasians and non-Caucasians, respectively. Within the 1-year following discharge for the ACS event, 24% of Caucasians and 21% of non-Caucasians had a prescription for a high-intensity statin, with a mean reduction in LDL-C from baseline of 11 mg/dL and 13 mg/dL, respectively. In the 12 months post-index, the mean number of follow-up encounters with a cardiologist was 7 among Caucasians and 5 among non-Caucasians and the mean number of in-person visits (any provider/visit type) was 19 and 16 among Caucasians and non-Caucasians respectively. 

Among 3367 (82.6%) urban and 709 (17.4%) rural patients, the mean age as of ACS was 68 and 65 respectively. The mean distance from a subject’s home was 12 miles among urban patients and 142 miles among rural patients. The mean baseline LDL-C was 87 mg/dL among urban patients and 91 mg/dL among rural patients. Rates of high-intensity statin prescriptions in the 1-year following the ACS event were 28% among urban patients and 25% among rural patients, with a mean reduction in LDLC from baseline to follow-up of 14 mg/dL and 16 mg/dL respectively. In the 12 months post-index, the mean number of follow up encounters with a cardiologist was 7 among urban patients and 5 among rural patients and the mean number of in-person visits (any provider/visit type) was 19 and 15 among urban and rural patients, respectively.

**Conclusions:** Within a large integrated health delivery network in northern Nevada, prescription of high-intensity statin treatment following a hospitalization for ACS is sub-optimal and does not vary by race or by urban/rural residence. This, in combination with the moderate baseline LDL-C levels prior to the ACS suggest that significant residual risk and gaps in care exist among this population of patients.

## 3. Perspectives

On 3–5 May 2019, CMHC will host its 3rd Annual CMHC West: Advancing Cardiometabolic Health from East to West, held in Phoenix. This two-day conference will capture the integrity and high-quality education of the annual Cardiometabolic Health Congress as the nation’s top experts in cardiometabolic health will highlight the latest updates in hypertension, heart failure, diabetes, lifestyle management and cardiovascular health. The most recent results from top-line clinical trials and new and emerging agents will be brought to you through thought-provoking and innovative education. 

For more information, please visit: https://www.cardiometabolichealth.org/2019/index-west.html.

